# A novel chitosan and polydopamine interlinked bioactive coating for metallic biomaterials

**DOI:** 10.1007/s10856-022-06688-x

**Published:** 2022-09-23

**Authors:** Erişen Deniz Eren, Gu Guisong, Liu Mingming, Zhang Bingchun, Yang Ke, Chen Shanshan

**Affiliations:** 1 School of Material Science and Engineering, University of Science and Technology of China, 230026 Hefei, China; 2 Shi-changxu Innovation Center for Advanced Materials, Institute of Metal Research, Chinese Academy of Sciences, 72 Wenhua Road, 110016 Shenyang, China; 3 University of Science and Technology of Liaoning, Anshan, China; 4 Shenyang University, 110044 Shenyang, China

## Abstract

Chitosan coatings have shown good bioactive properties such as antibacterial and antiplatelet properties, especially on blood-contacted biomedical materials. However, as blood-contacted biomedical device, the intravascular metal stent has a burden with adverse effects on the structural integrity, such as mechanical load during implantation and substrate degradation if a biodegradable metal is used as the substrate. It is unquestionably true that the structural integrity of the coated stent is essential. The adhesion strength between the coating and the substrate positively affects it. Silane and polydopamine (PDA) interstitial layers have been investigated to improve the corrosion resistance, biosafety and adhesion strength. This work addressed this challenge by using PDA as an intermediate and glutaraldehyde as a linking agent to establish a strong link between the polymer coating and the intermediate coating. Compared with PDA-only and glutaraldehyde-linked silane layer, the novel coating displayed a notable increase in adhesion. When compared with the bare Ni-free stainless steel, the performance of the novel coating was not significantly different. This novel chitosan film on the glutaraldehyde linked-PDA interface can be applied to various metallic substrates where synergic bioactive and anticorrosive effects of PDA interstitial coating and chitosan are needed.

**Figure:**
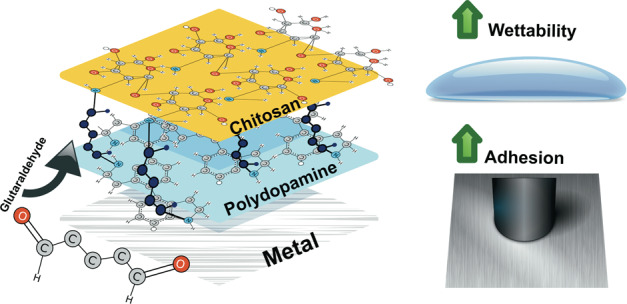
Graphical abstract

Graphical abstract

## Introduction

Chitosan is a polysaccharide produced by natural chitin alkaline deacetylated and can be sourced abundantly from nature, and most of them are produced from shrimp shells [1, 2]. It has up-and-coming properties and applications in structural biomaterials and medicine carrier materials. Chitosan and its composites and coating on devices have had a wide range of applications for drug delivery in the form of microspheres, tablets, nanoparticles, nanofibers, beads, films, and hydrogels [3–10]. Moreover, chitosan and its nanocomposite have more opportunities to be applied for drug-eluting biodevices [11, 12]. chitosan and chitosan composite coatings are also promising bioactive materials to design such properties as antibacterial and antiplatelet coatings on biomedical devices [13–20]. These properties are summarized in many studies as; supporting cellular activities (such as angiogenesis or thrombosis, etc.), delivering medical agents (such as drugs, cell cultures, etc.), and functional device components (such as biosensors, etc.) [21, 22]. Early research has already demonstrated that sulfated Sulfated N-(carboxymethyl) chitosan has the potential ability to be used as an anti-coagulation drug, and its anti-coagulation performance is as potent as heparin [23]. On the other hand, chitosan and its composites can be applied as a protective coating on biodegradable metallic biomaterials to slow down the degradation and withal to decrease possible toxicity [24–26].

Because structural integrity of any implant is an essential parameter, chitosan’s structure improvement and mechanical properties are also significant as a bioactive coating on biomedical materials and devices. Various methods can improve the mechanical properties of chitosan films [16, 19, 27–33]. Nevertheless, the surface properties of chitosan-coated bioactive substrates can be modified with various techniques. One of those options is using various grafting mechanisms of chitosan polymer to increase the structural integrity of substrate and coating [34]. For all those applications of chitosan on metal surfaces, silanization reaction can improve chitosan-metal surface attachment [17, 24, 35]. silane is a promising intermediate coating to increase adhesion while properly improving corrosion resistance or postponing biodegradable metallic materials [36]. Alternative to silane coating, polydopamine (PDA) has promising properties, forming a uniform film on the metal surface. It can also slow down the degradation rate of the metal substrate and reduce its potential toxicity [37]. Dopamine chemistry is also suitable for grafting bioactive coatings on stainless steel surfaces [38]. Moreover, dopamine chemistry is also available to build various linking mechanisms with chitosan polymer [39].

The structural integrity of polymer coating and the metal surface is essential for intravascular stents, which are stressed by many loads, particularly expansion deformation during angioplasty [40–42]. This process will create a deformation in the polymer coating. During this process, if polymer film on the metal surface lacks adhesion and plasticity, it will be shed from the metal substrate [43]. Therefore, the adhesion strength of the coating on a metal stent is a critical parameter besides the plasticity of the polymer film.

A novel chitosan coating process has been developed on various metallic surfaces for developing bioactive surfaces. Because both chitosan and dopamine monomers have primary amine functional groups which are ready to link with glutaraldehyde, due to double aldehyde functional groups glutaraldehyde, it can establish chemisorption between two interstitial layers contained amine groups. Glutaraldehyde was studied as the linking agent to establish bonding between PDA film and chitosan film.

## Experimental

### Materials preparation

**Fig. 1:**
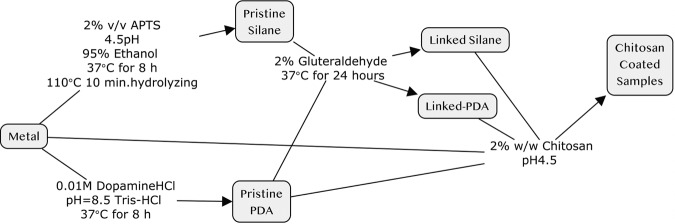
Coating steps

*Nickel-free stainless steel* (Ni-free-SS) was used as a rigid substrate to investigate the adhesion strength after various coatings. All the samples used for the adhesion strength test were ground by alumina papers up to 1000 grit and then were continued to be polished to 2000 grit with silicon carbide paper. Samples for contact angle measurement were subsequently polished by diamond paste. Ground Ni-free-SS substrates were cleaned for 30 min with 0.01 M citric acid solution, distilled water, absolute ethyl alcohol, acetone, and then absolute ethyl alcohol again ultrasonically. Polished samples were cleaned for 30 min at each step with distilled water, absolute ethyl alcohol, and acetone, and then absolute ethyl alcohol again ultrasonically. Coating steps of all samples are shown in Fig. 1.

#### PDA coating preparation

0.01 M Tris solution is prepared with distilled water and tris(hydroxymethyl)aminomethane salt (TRIS). Later its pH was configurated to 8.5 by titration with a small amount of hydrochloric acid (HCl) and sodium hydroxide (NaOH). First, coating samples are immersed in the solution. Later, 2 mg/mL 3-hydroxytryptamine hydrochloride (DopamineHCl) was added to the TRIS solution and mixed ultrasonically. During incubation, an excess amount of air contact with the reaction surface of samples at 37 °C for 8 h.

#### Silane coating preparation

95% ethyl alcohol solution has been prepared with pH 4.5 by adding some acetic acid and NaOH. Samples were immersed with the solution in petri dishes, and a silane coupling agent (3-Aminopropyl)triethoxysilane (APTS) was added to the solution at 2% volume percent of each solution. Samples were soaked inside APTS added solution at 37 °C for 8 h for silanization reaction. Then, the samples were washed gently with absolute ethyl alcohol and cured at 110 °C for 10 min for hydrolyzing.

### Aldehyde linked surface preparation

Silane-coated samples were denoted as group 1, and PDA-coated samples were denoted as group 2, then were soaked inside of 2% glutaraldehyde water solution at 37 °C for 24 h. While another PDA-coated sample was denoted as group 3, then just soaked in distilled water at 37 °C for 24 h. After soaking, glutaraldehyde-linked Silane (linked silane) and glutaraldehyde-linked polydopamine (linked-PDA) coated samples were washed with excess distilled water until all free glutaraldehyde was removed from their surfaces.

*Chitosan* solution preparation (1wt.%) is by 2% Acetic Acid solution, configured to pH4.5 by NaOH, similar to previous works [14]. Samples for the adhesion test are all coated with chitosan by solution casting method and dried at room temperature for 2 days under 20 relative humidity.

### X-ray photoelectron spectroscopy

Linked Silane, linked-PDA, and pristine-PDA grafted samples without chitosan polymer coatings, and a chitosan coating applied on the glutaraldehyde-linked dopamine coating were characterized by XPS (ESCALAB250, Thermo VG) to see radical groups at the outmost surface. In addition, an argon-ion beam etching system is applied to the linked-PDA sample. Then it is characterized on different etching levels by XPS to detect the depth profile of the coating. Etching calibration of the argon-ion beam was between 0.1 nm/s and 0.2 nm/s for a Ta_2_O_5_ film.

### Contact angle measurement

The contact angle of samples (θ_aq_ and θ_1BN_) with various coating systems were measured (TBU-95 DataPhysics, Germany) by sessile drops of deionized water and 1-bromonaphthalene (1BN). Then the polar components, dispersive components, and solid surface tensions of each different surface calculated by a method are shown by Formula 1 [44–46]. Critical surface tensions (γ_s_), components (γ_s_^p^, γ_s_^d^) of deionized water, and 1BN (see Table.1) were taken from previous works.

**Table 1:** Surface tension (r_s_), polar and dispersive components (r_s_^p^, r_s_^d^) of deionized water and 1BN

Reagents	γ_s_^p^	γ_s_^d^	γ_s_
Deionized water	51.0	21.8	72.8
1BN	0	44.6	44.6

1 $$\begin{array}{l}\gamma _{{{{\mathrm{aq}}}}}\left( {1 \,+\, \cos \theta _{{{{\mathrm{aq}}}}}} \right) \,=\, 2\left( {\gamma _{{{{\mathrm{aq}}}}}^{{{\mathrm{d}}}}\gamma _{{{\mathrm{s}}}}^{{{\mathrm{d}}}}} \right)^{1/2} \,+\, 2\left( {\gamma _{{{{\mathrm{aq}}}}}^{{{\mathrm{p}}}}\gamma _{{{\mathrm{s}}}}^{{{\mathrm{p}}}}} \right)^{{{{\mathrm{1/}}}}}\\ \gamma _{1{{{\mathrm{BN}}}}}\left( {1 \,+\, \cos \theta _{1{{{\mathrm{BN}}}}}} \right) \,=\, 2\left( {\gamma _{1{{{\mathrm{BN}}}}}^{{{\mathrm{d}}}}\gamma _{{{\mathrm{s}}}}^{{{\mathrm{d}}}}} \right)^{1/2} \,+\, 2\left( {\gamma _{1{{{\mathrm{BN}}}}}^{{{\mathrm{p}}}}\gamma _{{{\mathrm{s}}}}^{{{\mathrm{p}}}}} \right)^{1/2}\end{array}$$

### Adhesion strength measurement

Chitosan films on linked silane, linked-PDA, pristine-PDA bare Ni-free SS metals are tested by a pull-off test device (PosiTest Precious AT, Defelsko Corp. with The ResinLab EP11HT Adhesive Epoxy Resin) due to ISO 4624: 2002.

### Statistical analysis

Measured adhesion forces and wetting angles were analyzed using ANOVA at the 0.05 level, and the results are shown as mean ±1 standard error with minimum–maximum values. Fisher’s least significant difference test (LSD) and Tukey’s honest significant difference test (HSD) revealed significant differences between means.

## Results

### Spectroscopy

**Fig. 2:**
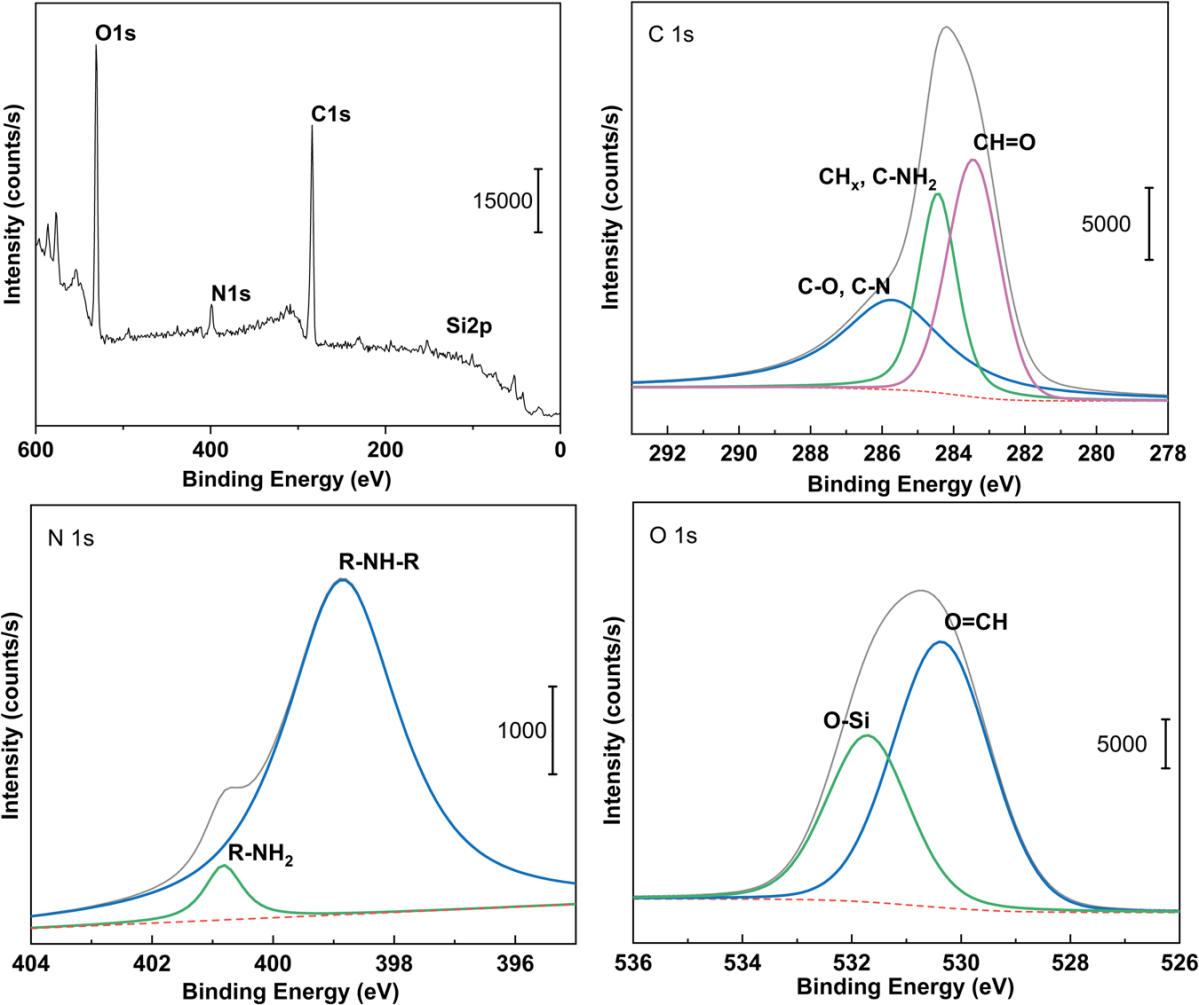
Surveys of linked silane XPS outmost layer and O1s, C1s, and N1s binding energy levels

Survey and the specific binding energies for O 1s, C 1s, and N 1s energy levels of silane samples are shown in Fig. 2. C–O–C groups, OH–C (carbon and oxygen single bonds)groups, O=CH (aldehyde) groups, R-NH_2_ (amine) groups, and R-NH-R (lactam) groups were fitted in each 1 s peak. The peak for O 1s of the aldehyde group was around 530.2 eV, and that of C 1s was 283.2 eV. The peak of the amine group was around 409.4 eV, and that of the lactam group was around 399 eV.

**Fig. 3:**
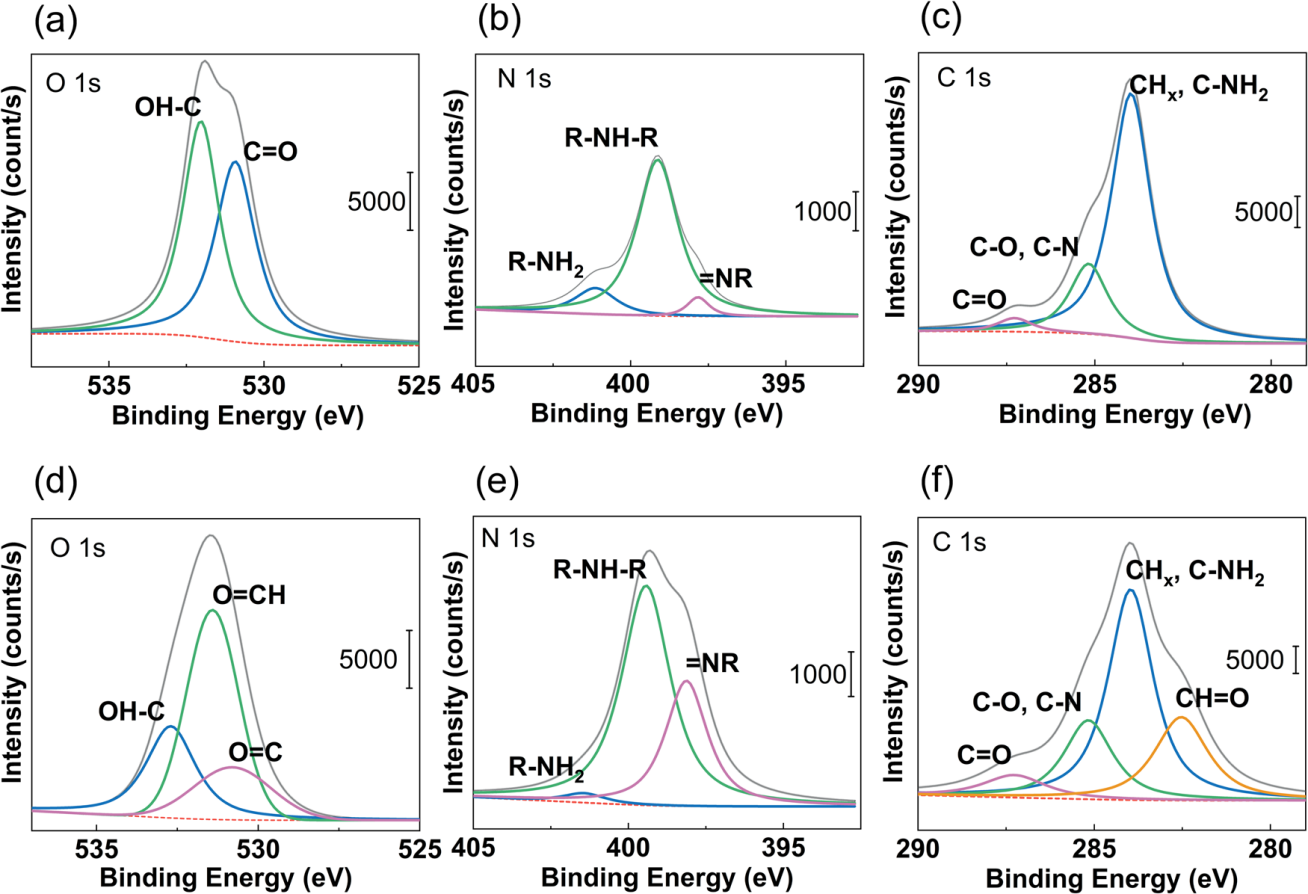
Binding energies of (**a**) O1s, (**c**) N1s, (**e**) C1s on the pristine-PDA surface and (**b**) O1s, (**d**) N1s, (**f**) C1s on the linked-PDA

**Fig. 4:**
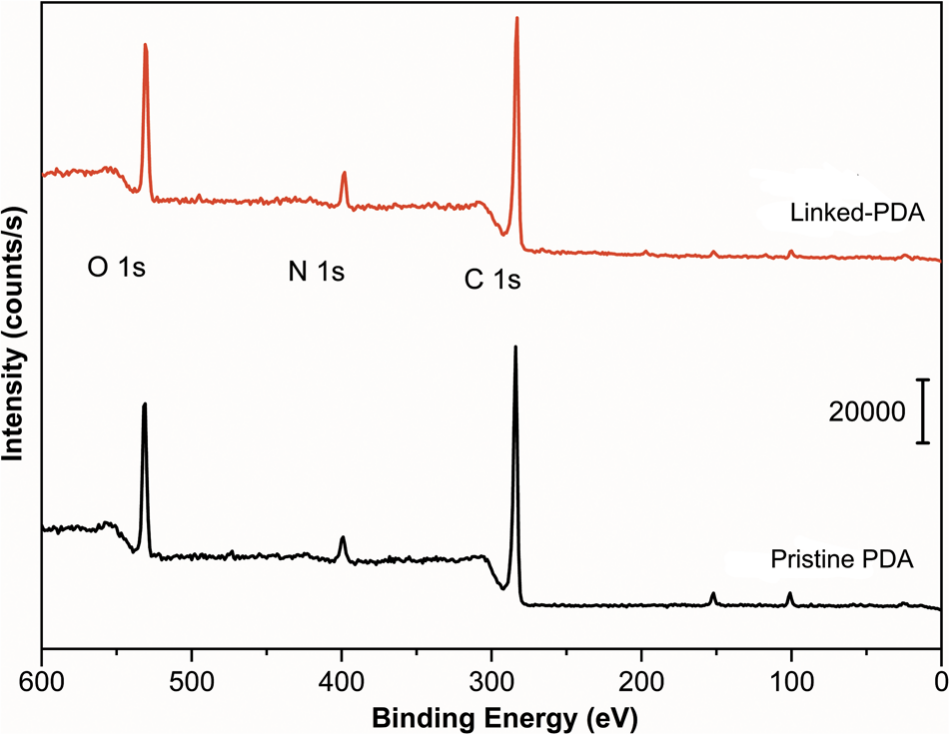
XPS surveys of linked (red line) and pristine (black line) PDA outmost layers

Specific binding energies for O1s, C1s, N1s energy levels of pristine-PDA and linked-PDA samples are shown in Fig. 3, while the survey binding energies are compared in Fig. 4. Carbon and oxygen single bonds, carbonyl groups, aldehyde groups, amine groups, =NR (imine) groups, and lactam groups are fitted in each 1s peak.

Figure 3a shows the O1s peak of the pristine PDA, while Fig. 3d shows the O1s peak of the linked-PDA. It can be seen that the glutaraldehyde treatment created some aldehyde groups on the surface. On the other hand, by comparison between Fig. 3b, e, it can be seen that the number of amine groups was decreased with glutaraldehyde treatment.

**Fig. 5:**
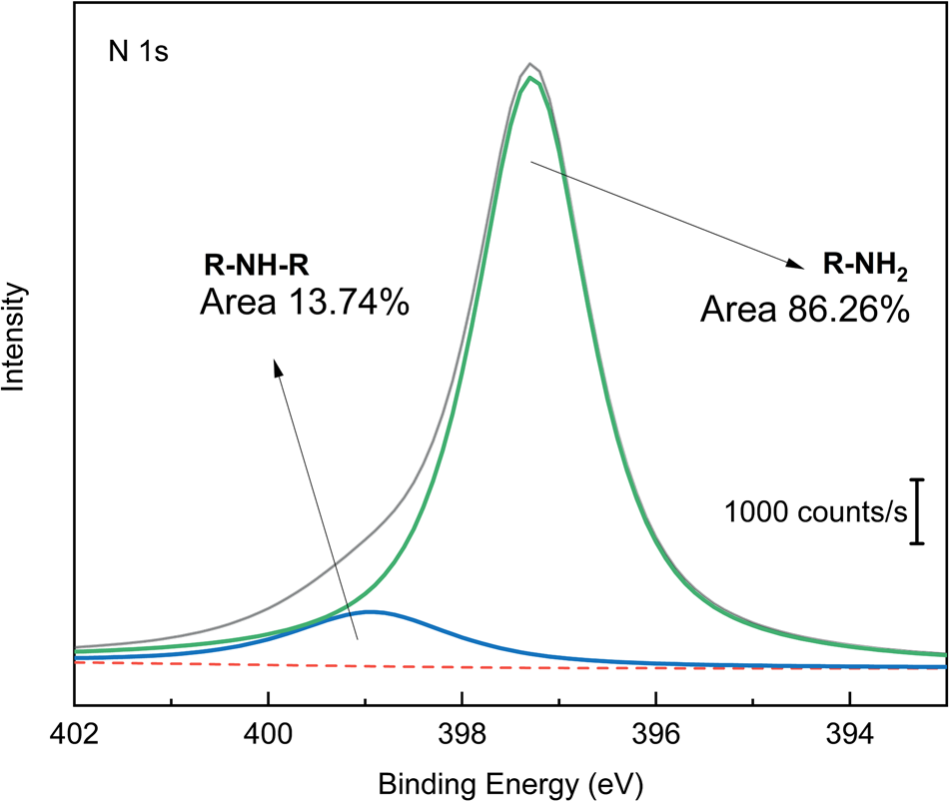
Binding energies of secondary amine (R-NH-R) and primary amine (R-NH2) bonds of chitosan film applied on the glutaraldehyde linked-PDA films

The Lactam and amine binding ratio of the polydopamine-chitosan interlinked sample is shown in Fig. 5. N1s peak shared with 86.26% amine groups and 13.74% lactam groups.

Results of linked-PDA samples etched for different times were collected as etching levels, where 10, 20, 30, 60, 80, 100, 120 s etched levels were named EL1, EL2, EL3, EL4, EL5, EL6, and EL7. The outmost surface level, which was not etched, was called L0. The etching level and atomic ratios of C, N, and O atoms at various levels are listed below in Table 2. It shows percentages of C, N, and O atoms in total organic matter. Level 0 with oxygen-rich groups had the highest amount of O, such as carbonyl and aldehyde. While reaching Level 4, the interface between metal and dopamine began to form, and O content increased relatively.

**Table 2:** Etching level, etch time, average depth and atomic ratios of C, O, and N elements at various etch levels from the surface

Etch Level	Etch Time (sn)	Average depth (nm)	Atomic percent^a^ of C (m/m%)	Atomic percent^a^ of O (m/m%)	Atomic percent^a^ of N (m/m%)
0	0	–	75.7914	18.5704	5.63821
1	10	2	84.7037	6.65797	8.63835
2	20	4	87.2613	6.11096	6.62773
3	30	6	90.157	5.18719	4.65582
4	60	12	86.2878	7.67838	6.03381
5	80	15	83.2602	10.6129	6.12682
6	100	20	82.9887	10.9041	6.10715
7	120	25	81.9744	10.2058	7.81983

^a^Molar percentage in total mol of C, O, N elements

**Fig. 6:**
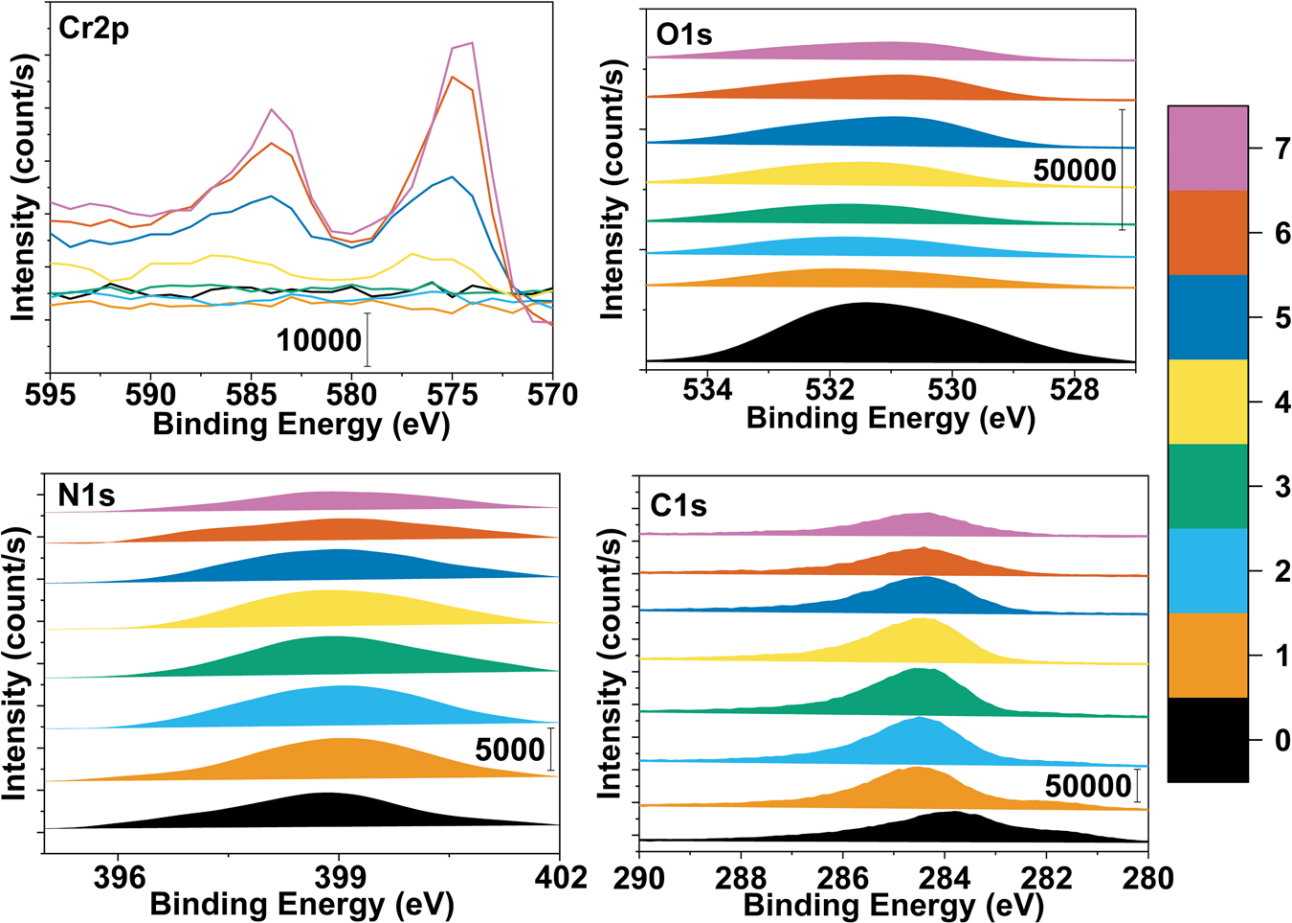
Layer by layer binding energies of the linked-PDA surface. Numbers represent the etching levels 0–7 which equal to 0, 10, 20, 30, 60, 80, and 120 s

Deep profiling of linked-PDA is presented in Fig. 6, showing every different etching level from 0 to 7, where etching times varied from 0 to 120 s. Etching level 0 is the outmost layer. While etching level increased on interstitial layers appeared, about 10 nm in depth. Level 0 peaks clearly show aldehyde groups on the surface. In comparison, EL4–EL7 peaks show a metal interface with increasing chrome content.

**Fig. 7:**
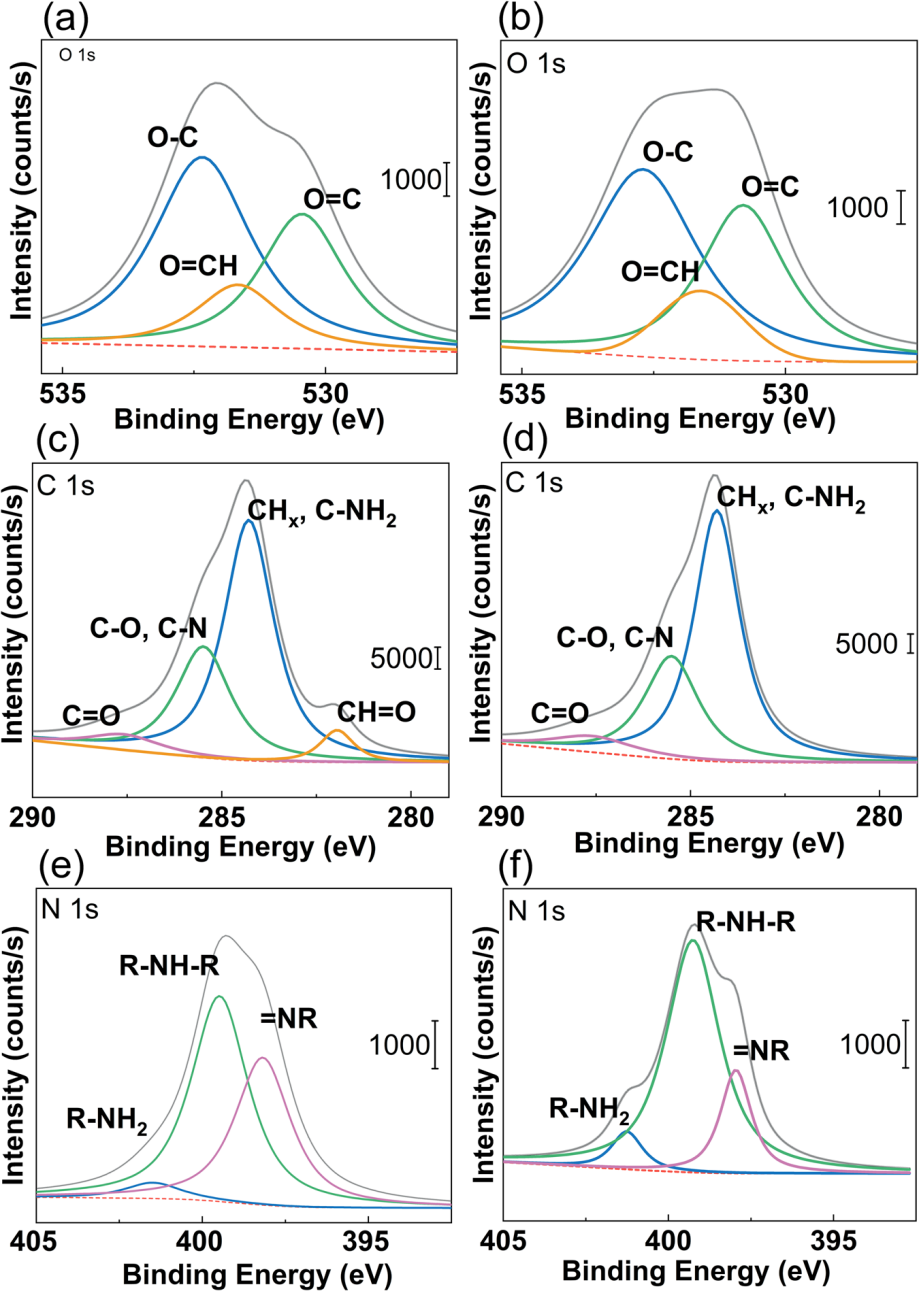
Binding energies of (**a**, **b**) O 1s, (**c**, **d**) C 1s (**e**, **f**) N 1s on the linked-PDA surface from (left) EL1 and (right) EL2

Fitted binding energy graphs of O 1s, N 1s, and C 1s regions of EL1 and EL2 samples are shown in Fig. 7. It is seen that the N 1s peak of the amine group, which had been decreased after glutaraldehyde treatment, was increased at EL2. On the other hand, the C 1s peak of aldehyde functional groups was decreased at EL1 and disappeared with EL2; consequently, the O 1s peak of aldehyde functional groups was slightly decreased by etching level increase. These phenomena can be described that glutaraldehyde reacting with the amine surface functional groups and creating linked lactam chains were ended with aldehyde surface functional groups, but the effect of glutaraldehyde was decreased by increasing the depth of linked-PDA film.

All peak positions and peak areas of PDA samples are listed in Table 3. O 1s peaks of carbon-oxygen single bond groups were fitted to be around 532.70–531.16 eV, and those of carbonyl groups were around 531.40–529.93 eV. N 1s peaks Amine groups were fitted to be around 401.48–401.16 eV, those of lactam groups were around 399.48–399.03 eV, and those of imine groups were around 398.18–397.73 eV. C 1s peaks of carbonyl groups were fitted to be around 287.59–286.24 eV, those of CH_x_ and C–NH_2_ groups were around 284.27–283.02 eV, and those of carbon-oxygen single and C–N groups were around 285.49–284.16 eV. Unless pristine PDA and EL2 of linked-PDA, all XPS curves had O 1 s peaks of aldehyde groups which were fitted to be around 531.66–531.40 eV. Ratios of peak areas were simply calculated by dividing the area into all areas of the regions.

**Table 3:** Peak positions and areas of fitted peaks of pristine polydopamine (PDA) samples and linked polydopamine samples with different etch levels (EL0, EL1, EL2)

Sample	Position (eV)	Area	Ratio	Functional Group
Pristine PDA	531.16	42915.16	57.84	OH–C
	529.93	31281.91	42.16	O=C
	401.16	1420.94	12.51	RNH_2_
	399.16	9136.04	80.45	R_2_NH
	397.86	799.20	7.04	N=R
	286.24	6313.74	6.44	C=O
	283.02	59480.70	60.66	CH_x_, C–NH_2_
	284.16	32254.54	32.90	C–O, C–N
Linked PDA (EL0)	532.70	19805.56	43.61	OH–C
	530.80	12805.56	28.20	O=C
	531.40	38050.86	28.20	O=CH
	401.03	746.90	5.63	RNH_2_
	399.03	8962.00	67.55	R_2_NH
	397.73	3559.22	26.83	N=R
	287.25	12749.55	7.29	C=O
	282.53	40522.51	23.16	CH=O
	283.95	86932.72	49.68	CH_x_, C–NH_2_
	285.15	34792.95	19.88	C–O, C–N
Linked PDA (EL1)	532.34	18408.48	52.53	O–C
	530.44	11408.48	32.55	O=C
	531.66	5227.69	14.92	O=CH
	401.48	974.73	4.24	RNH_2_
	399.48	12945.17	56.37	R_2_NH
	398.18	9046.28	39.39	N=R
	287.59	12272.92	5.63	C=O
	281.95	11432.30	5.25	CH=O
	284.29	128835.60	59.18	CH_x_, C–NH_2_
	285.49	65170.31	29.93	C–O, C–N
Linked PDA (EL2)	532.70	20285.72	53.46	O–C
	530.80	13285.72	35.01	O=C
	531.60	4376.44	11.53	O=CH
	401.256	1303.89	6.922	RNH_2_
	399.26	13970.21	74.17	R_2_NH
	397.96	3561.789	18.91	N=R
	287.57	16011.41	7.11	C=O
	284.27	131289.4	58.32	CH_x_, C–NH_2_
	285.47	74275.49	33.00	C–O, C–N

### Surface tension and wetting

**Fig. 8:**
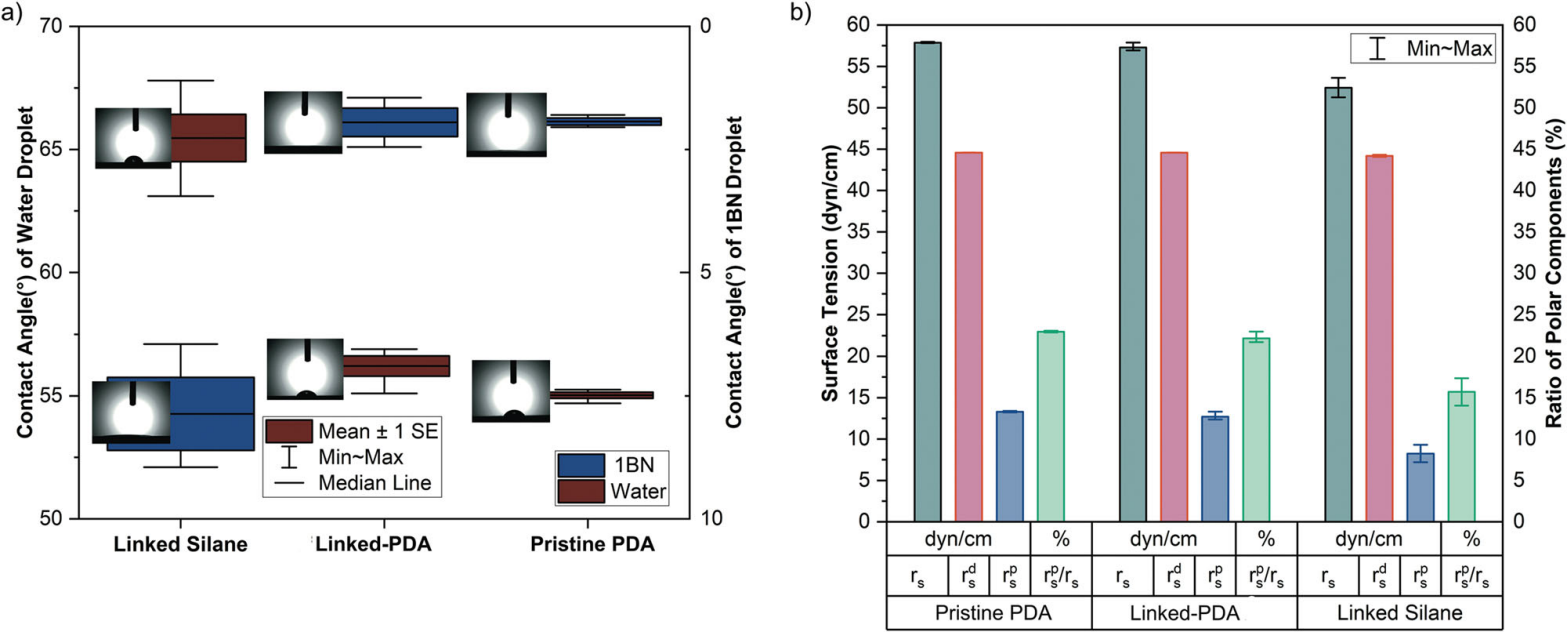
**a** Contact angles and (**b**) surface properties of pristine PDA, linked-PDA and linked silane

Wetting angles of three different samples are shown in Fig. 8a. Different grafted molecules to the surface showed mainly different results. Linked silane group samples showed significantly more hydrophobic properties, while linked-PDA and pristine-PDA interstitial coating coated surfaces showed hydrophilic properties compared to the silane-coated samples. Linked-PDA samples were slightly more hydrophobic than pristine PDA-coated samples. LSD revealed that the wetting angle difference between linked-PDA and pristine PDA was significant, but HSD revealed the difference was not significant.

The contact angle measurement with 1BN represented similar results. The contact angles of 1BN droplets on the glutaraldehyde-linked silane surface were almost three times higher than those on pristine dopamine samples. On the other hand, there is no significant difference between linked-PDA’s and pristine PDA’s contact angles.

1BN is a non-polar substance. It is used for the calculation of dispersive surface tensions. Later, polar components of surface tensions were calculated by the contact angles of sessile water droplets (Fig. 8b). Silane samples showed lower solid surface tension, while linked and not-linked dopamine samples showed similar solid surface tension. Polar components of silane-coated samples were lower than the dopamine-coated samples.

### Adhesion strength

**Fig. 9:**
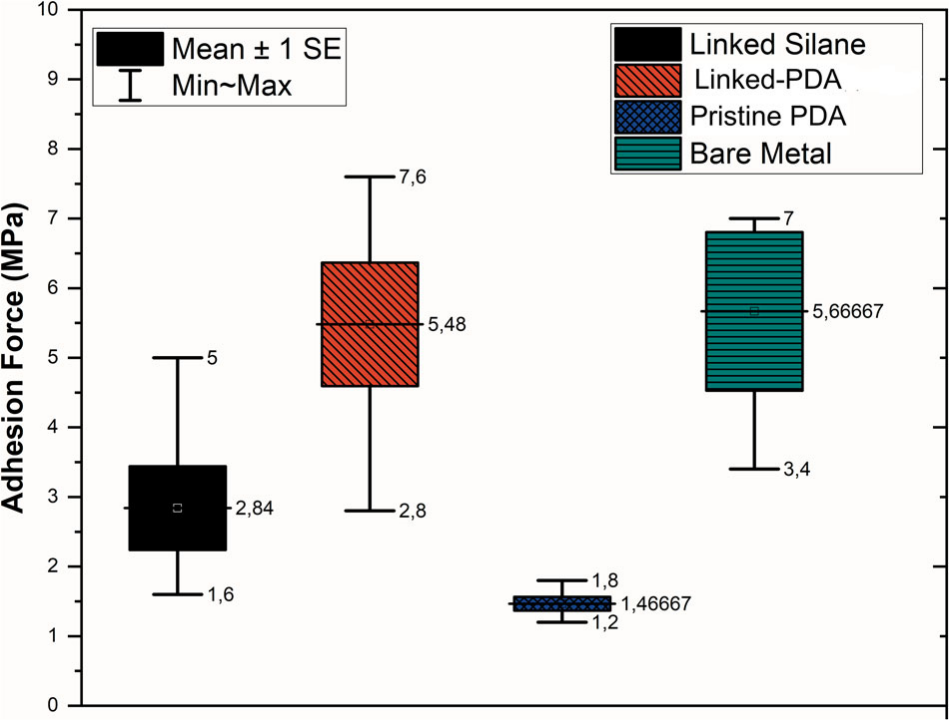
Adhesion strengths chitosan coatings on various interstitial layers coated (black and blue), linked (red) bare (green) Ni-free stainless steel

The pull-off test measured the adhesion strength of chitosan films to surfaces, and the results are shown in Fig. 9. The linked silane interstitial coating supplies 2.84 MPa binding force to the chitosan film, while the novel, linked-PDA interstitial coating showed a 5.48 MPa averagely. While pristine-PDA interstitial coating showed the poorest adhesion, 1.46 MPa on average, the bare stainless-steel samples showed similar adhesion to the novel coating, 5.66 MPa on average. Neither HSD nor LSD reveals a significant difference between the linked-PDA and bare-metal samples.

**Fig. 10:**
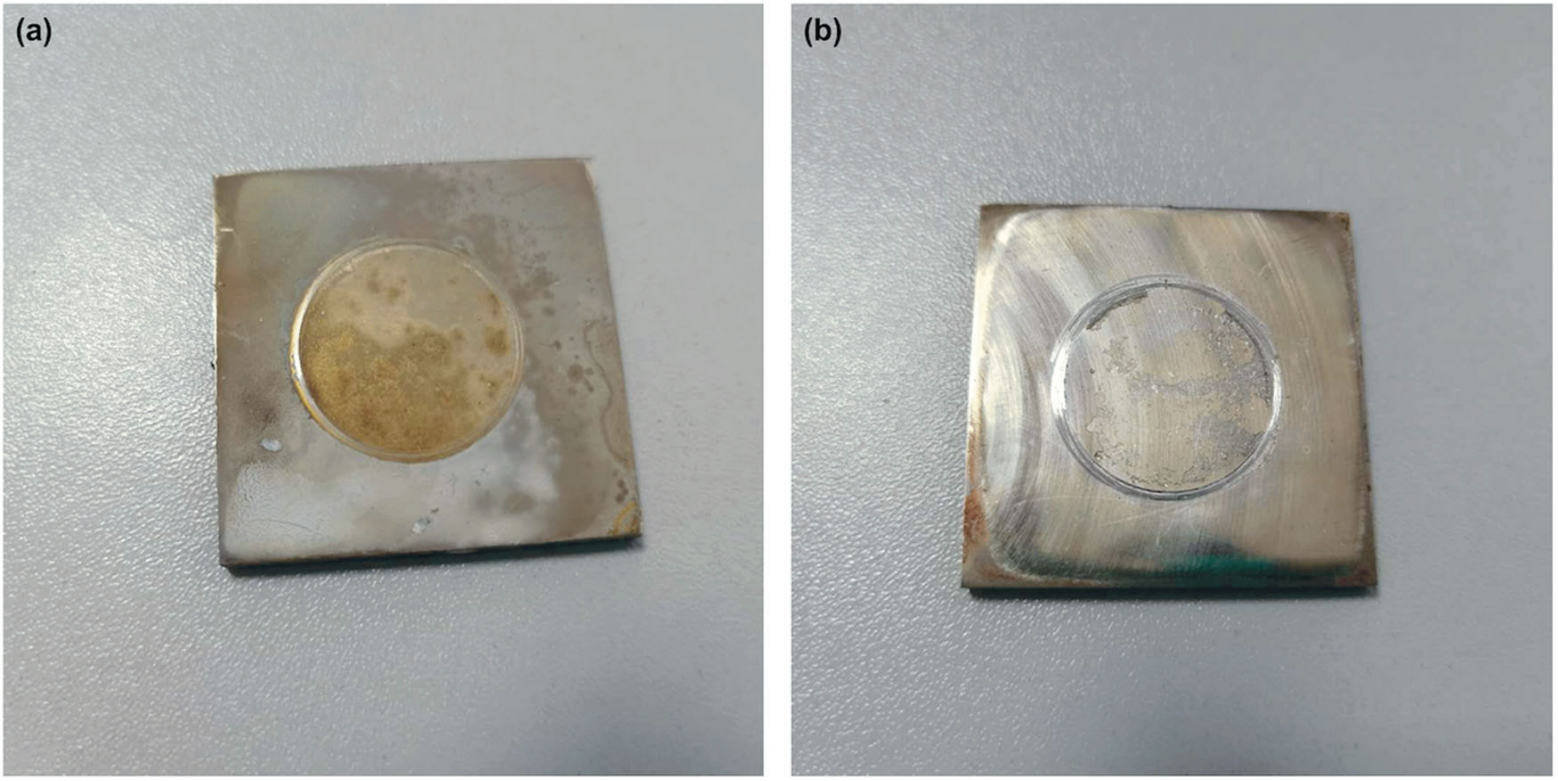
Macrographs of samples after adhesion strength measurement. **a** Pristine PDA and chitosan; (**b**) linked-PDA and chitosan

Two samples were photographed to show the difference between glutaraldehyde-linked coating and not-linked coating (Fig. 10). After the pull-of test, the chitosan film was utterly and uniformly pulled off from the pristine dopamine surface. On the other hand, chitosan film was not entirely and not uniformly pulled out from the linked-PDA surface, where the PDA film was pulled out together with chitosan.

## Discussion

Glutaraldehyde with double aldehydes functional group is known as a linking/crosslinking agent with organic materials of amine functional groups. It creates strong chemisorption between two interstitial layers. The study aimed to investigate a novel method to increase the adhesion force between chitosan and PDA interstitial layer to develop a bioactive chitosan-PDA film with high structural integrity. Glutaraldehyde-linked silane, glutaraldehyde linked-PDA, pristine PDA, and bare-metal surfaces have been investigated to (1) understand the chemical structure of glutaraldehyde-linked Silane, PDA, and chitosan, (2) find the adhesion performance of several interstitial layers to chitosan, and (3) understand the relationship between the specific surface tension of interstitial surfaces and the chitosan adhesion.

Linked silane, pristine PDA interstitial coating, and the bare-metal surface were compared with the performance of linked-PDA interstitial coating for the chitosan adhesion surface. Moreover, surface energies of interstitial layers were investigated to understand the wetting properties of overcast chitosan gel to form uniform chitosan films.

Firstly, spectroscopic investigations were held to understand the bond and layer structure of novel interstitial coating based on PDA and PDA linked to chitosan via glutaraldehyde. Spectroscopic results from different coating steps showed precise results about linking glutaraldehyde’s reaction to chitosan, PDA, and silane, respectively. N 1s results in Fig. 3 proved that the residual amine bonds after polymerization of dopamine had a vital role in the linking mechanism on the chitosan by glutaraldehyde linking agent. On the one hand, Fig. 3b yields similar results to previous characterization studies [47]. On the other hand, due to the novel method, the amine bond almost disappeared after glutaraldehyde treatment. As seen in Fig. 3, after treatment with glutaraldehyde, the PDA surface created lactam bonds. The spectroscopic result in Fig. 5 also proved that the lactam bond formation on chitosan after chitosan reacted with glutaraldehyde treated and free glutaraldehyde dopamine surfaces. Besides, after EL1, amine bonds were revealed. The N 1s peak of amine in Fig. 7d almost reached half value in Fig. 3b. While N 1 s peak area ratio of the pristine-PDA’s amine was 12.51%, and that of the EL2’s amine was 6.92%. Moreover, changes in the amine group alone were insufficient to prove the structural changes brought about by glutaraldehyde. However, if looking at the C 1s and O 1s binding energy regions, chemical changes could be seen in the PDA film after glutaraldehyde and the aldehyde functional groups were ready to form chemical bonds with chitosan. Aldehyde bonds were revealed after glutaraldehyde treatment, as seen in Fig. 3d, f. While the newly formed aldehyde functional groups had about 28.2% of the area in the O 1s band, reaching an area of approximately 23.06% similarly and roughly the same amount in the C 1s band, only because of the treatment with glutaraldehyde. Those percentages were based only on the ratio of the area occupied by the band in the XPS spectrum to the region where the band was located, and it was not sufficient to indicate the relative density of the chemical bonds formed. In short, it indicated how much the newly formed chemical bonds occupied the new state compared to each other in the previous and next states. To make a more accurate inference, we would have to know the relative binding energies of similar bonds under normal conditions and calculate accordingly. However, no database could make sufficient spectroscopic examinations of this novel bond structure, which had just been applied.

On the other hand, depth profiling with different etching levels represented different interstitial layers of the whole coating system of linked-PDA. As seen in Fig. 6, Levels 0–1 represent the highly glutaraldehyde-linked layer, where Levels 1–4 represent the interstitial dopamine layers that were affected hardly. Figure 6 and Table 2 show that Level 0 has the highest oxygen content due to the aldehyde functional group rich semi-linked glutaraldehyde layer, which was ready to react with chitosan coating is applied. When it reached Level 4, a stainless steel-dopamine interface was seen. The dopamine layer is dispersed into the passive oxide layer. Figure 6 and Table 2 show that O content increased after Levels 4 to 7. At this level, the XPS sector was not examined in terms of the formation of different bonds, especially considering both the passive surface of the metal and dopamine in the oxygen spectrum, as well as possible impurities and the new structural changes brought about by the novel method. Therefore, it would be impossible to make an accurate XPS analysis for each bond structure separately at this level. There could be two explanations that elemental results could be seen in the region from EL4 to EL7 to the extent that they were the results of both organic compounds and a passive inorganic film on the metal surface. The first was the possibility that the PDA grafted with the passive surface and the passive metal surface were not smooth enough. The second was the selective degradation of the passive inorganic film and the PDA organic film during etching. The second priority seems more likely due to the last finishing on the metal surface.

**Fig. 11:**
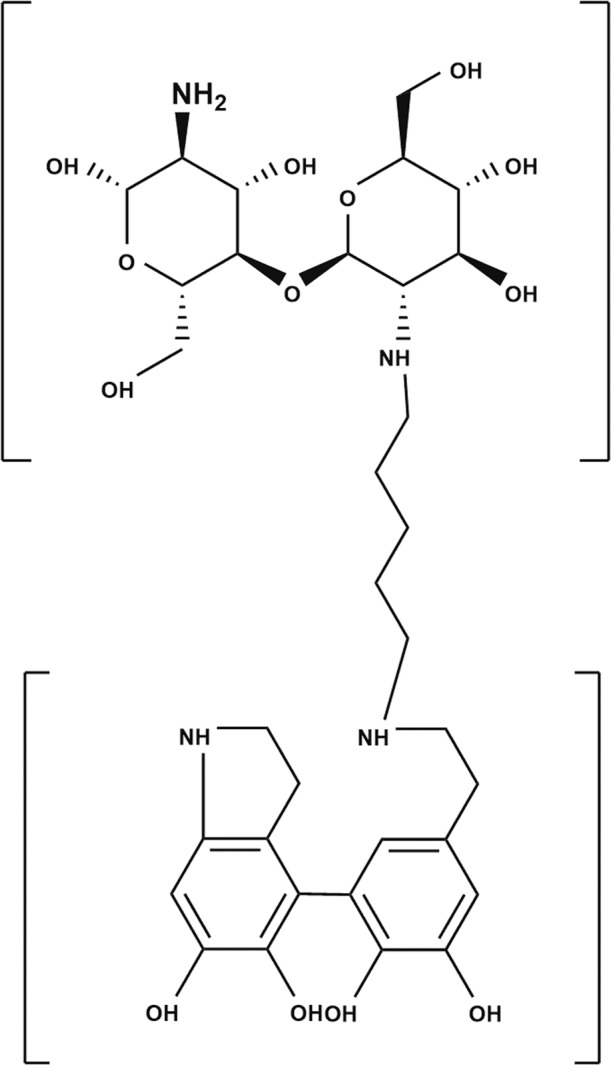
The schematic diagram of the molecular structure of glutaraldehyde linked-PDA-chitosan coating

Secondly, screening on the adhesion force of various interstitial layers to chitosan coating demonstrated the performance of a novel interstitial coating to other interstitial coatings. Adhesion force experiments showed that linked-PDA surfaces had a higher adhesion force to the chitosan film. The adhesion force of chitosan film to linked-PDA samples was double that of linked silane samples. Moreover, they were more than double the pristine dopamine. Double sides of glutaraldehyde aldehyde groups were responsible for this chemisorption. As molecular level explanation is described in Fig. 11, glutaraldehyde created a bond with chitosan and dopamine interstitial layers.

Thirdly, the surface properties of silane-coated and PDA-coated samples showed opposite properties. Linked silane was more hydrophobic than PDA-coated samples. At the same time, the wetting angle of silane-coated samples varied between 64° and 66° and PDA samples between 55° and 57°. Glutaraldehyde treated samples were slightly more hydrophobic than pristine dopamine-coated samples. This is undoubtedly very important for the polymer coating process and subsequent structural integrity made in an aqueous medium.

If looking at the specific surface tension, results of dopamine-coated samples possessed more promising surfaces to apply any water-soluble polymer coating on them due to the higher polar component of surface tension and lower hydrophobicity. Nevertheless, the linked-PDA-coated samples showed similar surface properties compared to the pristine PDA-coated samples, while their adhesion forces to chitosan layers were much higher than those of pristine PDA-coated samples. The higher experimental adhesion force on the linked-PDA surface was interlinked with glutaraldehyde treatment to chitosan, and a strong covalent bond between dopamine and glutaraldehyde was established. Contact angle measurements with water and 1BN only inspected polar and dispersive forces of the surface but not chemisorption. During this study wetting angle of chitosan solution and critical sliding angle of chitosan solutions were not investigated. It needs to be investigated to understand adhesion strength, besides the process conditions of chitosan coatings.

On the other hand, the polar component of the surface tension of dopamine-coated samples was calculated to be almost double the polar component of the surface tension of silane-coated samples. The higher γ^p^/γ^d^ ratio of the surface would give better wettability with chitosan coating, and hence chitosan also showed a high γ^p^/γ^d^ ratio [14].

Although the biodegradation of a chitosan film with a silane interlayer coating against an interlayer film with a silane dopamine interlayer coating cannot be inferred from these results, the effect of hydrophobic and hydrophilic properties of both interlayers on the degradation of body fluid should be investigated in the future studies. However, considering the process conditions and the mechanical loads during implantation, the linked-PDA chitosan coating obtained by the novel method has a tremendous advantage, confirmed in the adhesion results.

## Conclusion

This study clearly showed that the glutaraldehyde treatment on the PDA surface increased the adhesion strength of chitosan to the surface by creating a link via reaction with both amine groups of the chitosan and PDA, which can be called chemisorption. While this grafting mechanism is similar to the previous work between silane and chitosan [17], it showed a more potent adhesion force than the linked silane coating for chitosan film applications and better wettability for coating. While compared to the bare-metal chitosan coating, the adhesion strength of glutaraldehyde linked-PDA-chitosan coating on metal is not significantly different, and nevertheless, due to the bifunctionality of the dopamine coating on different metal surfaces, it has more opportunity compared to bare-metal chitosan coating.

## Supplementary Information

Supplementary Information
