# Supplementary material for: A novel chitosan and polydopamine interlinked bioactive coating for metallic biomaterials
Source: J Mater Sci Mater Med. 2022 Sep 23;33(10):65. doi: 10.1007/s10856-022-06688-x (PMC9499904; doi:10.1007/s10856-022-06688-x)
Supplement: Supplementary file 1 — Supplementary Information [file 10856_2022_6688_MOESM1_ESM.docx]

Electronic Supplementary Document

Statistical analysis

ANOVA is a very important method in testing whether there is a statistically significant difference between the means of independent groups. In addition to these statistical results with OriginPro, Tukey's HSD and Fisher's LSD tests, in which each paired group was compared with each other, were also used.

The one way ANOVA test did not show us between which groups the difference was. Therefore, we turned to post-hoc tests to obtain this information. These tests are useful for determining between which groups the difference detected in the ANOVA test. On the other hand, post-hoc tests mean nothing if ANOVA doesn't indicate a difference. Even if post hoc tests indicate a difference between some groups, we do not take them into account. For this reason, a difference was sought in the ANOVA results first. post-hoc tests were performed after the absolute result was positive. Two different post-hoc tests were used, Tukey's HSD and Fisher's LSD.

- 1. One-way ANOVA results of adhesion strength test samples

One-way ANOVA have been applied with OriginPro. Analysis gives results of described statistic and overall ANOVA result. Mean, SD and SE values are shown in the Table A-1 Descripted statistic of adhesion strength measurement below.

Table A-1 Descripted statistic of adhesion strength measurement

|  | Mean | Standard Deviation | SE of Mean |
| --- | --- | --- | --- |
| Linked Silane | 2.84 | 1.34462 | 0.60133 |
| Linked PDA | 5.48 | 1.98293 | 0.88679 |
| Pristine PDA | 1.46667 | 0.24221 | 0.09888 |
| Bare Metal | 5.66667 | 1.97315 | 1.1392 |

Due to Table A-2 Overall ANOVA results of adhesion strength samples below the samples’ means are significantly different. (P<0.005). Then we can apply ad-hoc significant different test to se whether the means are significantly different each other where the significance were compared by one by.

Table A-2 Overall ANOVA results of adhesion strength samples

|  | DF | Sum of Squares | Mean Square | F Value | Prob>F |
| --- | --- | --- | --- | --- | --- |
| Model | 3 | 60.62737 | 20.20912 | 9.76601 | 8.06078 10^-4^ |
| Error | 15 | 31.04 | 2.06933 |  |  |
| Total | 18 | 91.66737 |  |  |  |

The homogeneous distribution of the measurements is also important. Non-homogeneous measurements may be evidence of arbitrary measurements or the presence of a second factor that systematically influences the results. Levene’s test revealed measurement’s variances are significantly different (P<0.005). Details are shown in the Table A-3 Absolute deviations, homogeneity of variance test of adhesion strength samples below.

Table A-3 Absolute deviations, homogeneity of variance test of adhesion strength samples

|  | DF | Sum of Squares | Mean Square | F Value | Prob>F |
| --- | --- | --- | --- | --- | --- |
| Model | 3 | 6.32435 | 2.10812 | 5.00026 | 0.01337 |
| Error | 15 | 6.32403 | 0.4216 |  |  |

- 1. Post-hoc results of adhesion strength test samples

By looking at the results of ANOVA and Levene's absolute deviation before, we understood that the measurements were significantly distributed in total. However, this does not mean that measurements made in two different sample groups should be compared with each other as large or small. Tukey's HSD method and Fisher's LSD method were used to find out whether the sample groups were compared one by one and gave average different results from each other.

- - 1. Tukey’s HSD

Tukey's HSD method is the most sane comparison method that gives the most academic results. Linked PDA, linked silane. When we look at the adhesion strength measurements of pristine PDA and chitosan coating on bare metal, we see a wide distribution of results for each sample group. The values obtained from the ANOVA results are replaced in Tukey's method and it is examined whether the mean values of these distributions are comparable with each other as shown in the Table A-4 Tukey’s HSD comparisons of adhesion strength samples. In the comparison, it was predicted statistically that a comparison between Pristine PDA versus linked silane and bare metal versus linked silane and bare metal versus linked PDA would not be significant. It was stated that other comparisons could be made as significant as the difference between their mean.

Table A-4 Tukey’s HSD comparisons of adhesion strength samples

| Factor | MeanDiff | Error | Significant? |
| --- | --- | --- | --- |
| Linked-PDA vs Linked Silane | 2.64 | 2.622161 | Yes |
| Pristine PDA vs Linked Silane | -1.37333 | 2.510528 | No |
| Pristine PDA vs Linked-PDA | -4.01333 | 2.510528 | Yes |
| Bare Metal vs Linked Silane | 2.826667 | 3.027811 | No |
| Bare Metal vs Linked-PDA | 0.186667 | 3.027811 | No |
| Metal vs PDA | 4.2 | 2.931665 | Yes |

In this case, it can be said that linked PDA provides higher adhesion than linked silane and pristine PDA, as discussed in the results.

- - 1. Fisher’s LSD

Fisher's LSD, as the name suggests, can make sense of the smallest differences. In this respect, it is a more liberal method, but observation can be used to reveal small differences. As shown in the Table A 5 Fisher’s LSD comparisons of adhesion strength samples,

Table A 5 Fisher’s LSD comparisons of adhesion strength samples

| Factor | MeanDiff | Error | Significant? |
| --- | --- | --- | --- |
| Linked-PDA vs Linked Silane | 2.64 | 1.93919 | Yes |
| Pristine PDA vs Linked Silane | -1.37333 | 1.856633 | No |
| Pristine PDA vs Linked-PDA | -4.01333 | 1.856633 | Yes |
| Bare Metal vs Linked Silane | 2.826667 | 2.239183 | Yes |
| Bare Metal vs Linked-PDA | 0.186667 | 2.239183 | No |
| Metal vs PDA | 4.2 | 2.16808 | Yes |

this time bare metal and linked silane samples are revealed significant also.

- 1. One-way ANOVA results of contact angle results

Similarly Mean, SD and SE values are shown in the Table A-6 Descripted statistic of 1-HBN sessile drop measurement and Table A-7 Descripted statistic of water sessile drop measurement.

Table A-6 Descripted statistic of 1-HBN sessile drop measurement

|  | Mean | Standard Deviation | SE of Mean |
| --- | --- | --- | --- |
| Linked Silane | 12.025 | 7.8396 | 2.77172 |
| Linked `PDA | 2.0625 | 0.50125 | 0.17722 |
| Pristine PDA | 1.98 | 0.18135 | 0.05735 |

Table A-7 Descripted statistic of water sessile drop measurement

|  | Mean | Standard Deviation | SE of Mean |
| --- | --- | --- | --- |
| Linked Silane | 70.64167 | 7.8039 | 2.25279 |
| Linked `PDA | 55.05833 | 1.93365 | 0.5582 |
| Pristine PDA | 46.76667 | 12.25504 | 3.53773 |

And, due to Table A-8 Overall ANOVA results of 1HBN contact angles measurements and the Table A-9 Overall ANOVA results of water wetting angles measurements , means are significantly different. (P<0.005).

Table A-8 Overall ANOVA results of 1HBN contact angles measurements

|  | DF | Sum of Squares | Mean Square | F Value | Prob>F |
| --- | --- | --- | --- | --- | --- |
| Model | 2 | 554.79987 | 277.39993 | 14.75976 | 7.51937E-5 |
| Error | 23 | 432.26975 | 18.79434 |  |  |
| Total | 25 | 987.06962 |  |  |  |

Table A-9 Overall ANOVA results of water wetting angles measurements

|  | DF | Sum of Squares | Mean Square | F Value | Prob>F |
| --- | --- | --- | --- | --- | --- |
| Model | 2 | 3526.43056 | 1763.21528 | 24.62294 | 2.85822E-7 |
| Error | 33 | 2363.085 | 71.60864 |  |  |
| Total | 35 | 5889.51556 |  |  |  |

- 1. Post-hoc results of contact angle results

Likewise, both measurements and all three different groups were seen as factors and continued to be compared.

- - 1. Tukey’s HSD

As seen in the Table A 10 Tukey’s HSD result of 1HBN contact angle measurement , that is revealed, there is no significant difference between the wetting angle of 1HBN on the surface of pristine PDA and linked PDA

Table A 10 Tukey’s HSD result of 1HBN contact angle measurement

| Factor | MeanDiff | Error | Significant? |
| --- | --- | --- | --- |
| Linked-PDA vs Linked Silane | -9.9625 | 5.428403 | Yes |
| Pristine PDA vs Linked Silane | -10.045 | 5.149835 | Yes |
| Pristine PDA vs Linked-PDA | -0.0825 | 5.149835 | No |

As seen in the Table A-11 Tukey’s HSD comparison of water wetting angle measurement. Similar comparison is acceptable for wetting angles.

Table A-11 Tukey’s HSD comparison of water wetting angle measurement

| Factor | MeanDiff | Error | Significant? |
| --- | --- | --- | --- |
| Linked-PDA vs Linked Silane | -15.5833 | 8.476952 | Yes |
| Pristine PDA vs Linked Silane | -23.875 | 8.476952 | Yes |
| Pristine PDA vs Linked-PDA | -8.29167 | 8.476952 | No |

- - 1. Fisher’s LSD

As seen in the Table A-12 Fisher’s LSD comparison of 1HBN contact angle measurement that is revealed again, there is no significant difference between the wetting angle of 1HBN on the surface of pristine PDA and linked PDA

Table A-12 Fisher’s LSD comparison of 1HBN contact angle measurement

| Factor | MeanDiff | Error | Significant? |
| --- | --- | --- | --- |
| Linked-PDA vs Linked Silane | -9.9625 | 4.484067 | Yes |
| Pristine PDA vs Linked Silane | -10.045 | 4.25396 | Yes |
| Pristine PDA vs Linked-PDA | -0.0825 | 4.25396 | No |

On the other hand, slight significance is revealed between the hydrophobicity of pristine PDA and linked PDA.

Table A-13 Fisher’s LSD comparison of water wetting angle measurement

| Factor | MeanDiff | Error | Significant? |
| --- | --- | --- | --- |
| Linked-PDA vs Linked Silane | -15.5833 | 7.028587 | Yes |
| Pristine PDA vs Linked Silane | -23.875 | 7.028587 | Yes |
| Pristine PDA vs Linked-PDA | -8.29167 | 7.028587 | Yes |

- 1. Discussion

In the light of all these statistical methods, it should be known that there was no deficiency in the power size of the measurements made. However, the following implications from the results have been adequately discussed in the relevant chapters. (powers are bigger than 0.98)
